# The combination of high‐frequency QRS and ST‐segment alterations during exercise stress tests enhanced the diagnostic efficacy for coronary artery disease

**DOI:** 10.1002/clc.24254

**Published:** 2024-03-13

**Authors:** Long Liu, Xinyue Du, Xue Wei, Wei Dong, Hong Lu, Guishen Jiang, Guolan Deng

**Affiliations:** ^1^ The First Clinical College Chongqing Medical University Chongqing China; ^2^ Cardiovascular Medicine Department The First Affiliated Hospital of Chongqing Medical University Chongqing China; ^3^ School of Medical Imaging Zunyi Medical University Zunyi Guizhou China

**Keywords:** coronary artery disease, electrocardiogram, exercise stress test, high‐frequency QRS

## Abstract

**Background:**

High‐frequency QRS (HF‐QRS) manifests as a novel adjunct electrocardiographic marker with potential utility in coronary artery disease (CAD) detection.

**Hypothesis:**

We hypothesize that HF‐QRS analysis may be superior to conventional ST‐segment analysis in detecting CAD, and the combination of these two analyses in the exercise stress test may enhance the diagnostic efficacy for CAD.

**Methods:**

The study incorporated a sample of 157 patients (mean age 62 ± 9 years) referred for nonemergent angiography. Before angiography, patients underwent exercise stress testing utilizing an upright bicycle. High‐resolution electrocardiogram (ECG) data were collected during the exercise test, facilitating both HF‐QRS and conventional ST‐segment analyses. The diagnostic efficacy of HF‐QRS and ST‐segment analysis were compared, utilizing angiographic outcomes as the gold standard. The study design integrated HF‐QRS analysis and ST‐segment analysis via sequential and concurrent testing protocols.

**Results:**

In terms of CAD detection, HF‐QRS analysis displayed superior sensitivity compared to conventional ST‐segment analysis (63% vs. 37%, *p* = .002). The serial test significantly increased specificity from 79% to 97% (*p* = .002) compared to ST‐deviation analysis alone. It showed a markedly low sensitivity of 26%. The parallel test significantly increased sensitivity from 37% to 77% (*p* < .001), while retaining a moderate level of specificity of 51%. The quantity of ECG leads exhibiting a positive HF‐QRS response demonstrated a correlation with the severity of CAD (*p* < .001).

**Conclusions:**

HF‐QRS analysis exhibited superior sensitivity in detecting angiographically confirmed CAD relative to conventional ST‐segment analysis. Moreover, the combination of HF‐QRS and ST‐segment alterations during exercise stress test enhanced the diagnostic efficacy for CAD.

## Graphical abstract

High‐frequency QRS (HF‐QRS) manifests as a novel adjunct electrocardiographic marker with potential utility in coronary artery disease (CAD) detection. The authors find that HF‐QRS analysis is superior to conventional ST‐segment analysis in detecting CAD and the combination of HF‐QRS and ST‐segment alterations during exercise stress test enhances the diagnostic efficacy for CAD.

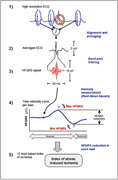

**Abbreviations**
CAD: coronary artery disease
CAG: coronary angiography
ECG: electrocardiogram
EST: electrocardiogram stress testing
HF‐QRS: high‐frequency QRS
MPHR: maximum age‐predicted heart rate
MPI: nuclear myocardial perfusion imaging

## 1 INTRODUCTION

Coronary artery disease (CAD) is a cardiovascular disorder typified by substantial impasses or blockages in the coronary arteries, primarily a consequence of atherosclerotic plaque accumulation. This may culminate in myocardial ischemia, hypoxia, or necrosis. An authoritative document demonstrates a 92.99% escalation in the global prevalence of cardiovascular diseases between 1990 and 2019, situating cardiovascular disease mortality as the leading cause of death globally.^1^ More specifically, ischemic heart disease and stroke are identified as the primary drivers of worldwide mortality and significant contributors to disability.^1^ Given the array of evidence‐based treatment options available, the early detection and risk stratification of CAD become crucial objectives within the medical field.^2^

The currently accepted noninvasive diagnostic examinations for patients suspected of myocardial ischemia include exercise electrocardiogram stress testing (EST), stress echocardiography, stress nuclear myocardial perfusion imaging (MPI), and coronary computed tomography angiography.^2^ Nevertheless, research suggested that EST displays a sensitivity range of 33%–50% and specificity between 77% and 89%, thus limiting its clinical efficacy.^3^, ^4^, ^5^, ^6^ In contrast, imaging techniques exhibit enhanced diagnostic accuracy. Studies indicate that supplementing exercise testing with MPI or echocardiography can boost sensitivity by 10%–20% and specificity by 10%.^7^ However, it is imperative to consider that specific imaging examinations expose patients to ionizing radiation, thereby underscoring the need for advanced noninvasive diagnostic technologies.

High‐frequency components of the QRS complex (HF‐QRS) offer a novel supplementary electrocardiographic marker with potential utility in CAD and ischemia detection.^8^, ^9^, ^10^ Conventional electrocardiogram (ECG) analysis during EST chiefly centers around ST‐depression as an index of altered repolarization. However, myocardial ischemia can also provoke depolarization disturbances, which are detectable and quantifiable through HF‐QRS analysis.^11^, ^12^ The standard ECG filters waveforms with frequencies ranging from 0.05 to 100 Hz. In contrast, HFQRS analysis, usually conducted in the 150 to 250 Hz frequency range, quantifies subtle changes in the propagation of the depolarization wavefront across the myocardium. Investigations involving human subjects have evidenced the superiority of HF‐QRS analysis over conventional ST‐segment analysis.^13^, ^14^ Integration of these two methodologies enhances diagnostic accuracy for myocardial ischemia.^15^ In these research endeavors, MPI served as the benchmark for ischemia, providing a physiological representation of CAD. Nonetheless, its correlation with anatomical CAD, as identified by coronary angiography (CAG), is not very satisfactory.^16^ There is a current scarcity of studies evaluating the diagnostic performance of HF‐QRS in patients with suspected CAD using CAG as an anatomical reference standard. Consequently, our study aimed to compare the effectiveness of HF‐QRS, conventional ST‐segment analysis, and a combination of these two analyses in the exercise stress tests for diagnosing CAD with CAG as the definitive benchmark.

## 2 METHODS

### 2.1 Population and protocol

In this forward‐looking investigation, we systematically enlisted 239 patients who were referred for nonemergency CAG. This process took place within the Department of Cardiology at The First Affiliated Hospital of Chongqing Medical University, China, during the period from November 2022 to June 2023. The decision to conduct CAG was specifically dictated by clinical indicators and remained independent of the study protocol. Before CAG, all patients were subjected to an upright bicycle exercise stress test. During the exercise test, continuous standard 12‐lead ECG was persistently recorded with an ECG SE‐1515‐Edan (Edan) and subsequently used for offline quantitative evaluation of ST‐segment deviations and HF‐QRS analysis. The exclusion criteria incorporated any contraindications to exercise testing, the presence of a cardiac pacemaker, atrial fibrillation, sustained ventricular arrhythmia during testing, left bundle‐branch block, complete right bundle‐branch block, or a QRS duration of 120 ms or more. Patients who were unable to reach a minimum of 80% of their maximum age‐predicted heart rate (MPHR) during the exercise test were excluded from further analysis. The study was conducted in accordance with the principles of the Declaration of Helsinki and received approval from the Ethics Committee of the First Affiliated Hospital of Chongqing Medical University. Written informed consent was obtained from all participants.

### 2.2 HF‐QRS analysis

HF‐QRS analysis was carried out in an automated and blinded manner using dedicated software (HyperQ™ Analyzer‐Stress, software version 2.2.1; BSP Ltd.).^13^, ^14^ The fundamental principles of this analysis are shown in Supporting Information S1: Figure 1. To summarize, using standard ECG methods to detect QRS complexes based on the signal amplitude (i.e., voltage). Noisy and ectopic beats were removed through dynamic template matching. The QRS complexes were averaged in each lead to produce a single representative QRS complex, thereby minimizing noise, with a default noise level threshold of 1 µV being employed. The averaged QRS complexes underwent digital filtration via finite impulse response filters to carry out band‐pass signal filtration within the 150–250 Hz range. Temporal trends of the HF‐QRS root‐mean‐square intensity throughout the exercise test were derived, and the reduction of HF‐QRS intensity (difference between the minimum and maximum HF‐QRS intensity) was calculated and used as an index of myocardial ischemia. The essential diagnostic criteria for a positive HF‐QRS response included adequate signal quality, an absolute reduction of ≥1 µV, and a relative reduction of ≥50% between maximum and minimum values in at least three leads. Patients with two or fewer leads exhibiting either HF‐QRS ischemia or excessive noise were classified as having a negative result. The HF‐QRS response was considered nondiagnostic if the HF‐QRS data did not indicate ischemia and three or more leads displayed high noise levels. These patients were subsequently excluded from the analysis.

### 2.3 Assessment of conventional ECG and CAG

The appraisal of the conventional ECG was executed by a proficient electrocardiographer, following established studies and guidelines meticulously. The process involved the use of real‐time ECG recorded at any juncture during the stress test and the automated ST‐measurements derived from the computer‐averaged ECG at distinct stages. Importantly, the evaluators were blinded to the CAG results, the HF‐QRS analysis, and clinical and exercise parameters. A positive EST result was defined by the emergence of a new horizontal or downsloping ST‐depression with an amplitude of at least 0.1 mV. This depression needed to endure for a minimum of 60–80 ms subsequent to the J‐point and be evident in two or more leads at any stage during the EST.^2^, ^7^, ^17^ Patients manifesting nonspecific or ambiguous ST changes were classified as inconclusive, and for data analysis, these “inconclusive” interpretations were deemed negative.

CAG was employed as the reference standard for comparison. The presence of CAD was confirmed when a stenosis surpassing 50% was detected in coronary arteries with a diameter of 2 mm or more, ascertained through visual interpretation.

### 2.4 Combined test

A composite test represents a method that combines different research tests to enhance diagnostic efficacy. There are two primary categories: series tests and parallel tests. In a series test, multiple tests are conducted in a sequential manner. A positive outcome in the preceding test triggers the immediate execution of the subsequent test; conversely, a negative outcome terminates the testing, thereby boosting specificity and diminishing misdiagnosis. In parallel testing, multiple tests are conducted concurrently, and a positive outcome in any test confirms the diagnosis, thereby enhancing sensitivity and reducing missed diagnoses. The study incorporated HF‐QRS analysis and ST‐segment analysis for CAD detection using both a serial test (where both HF‐QRS and ST‐segment analyses must be positive for a positive outcome) and a parallel test (where a positive outcome is achieved if either HF‐QRS analysis or ST‐segment analysis is positive).

### 2.5 Statistical analysis

Continuous variables are depicted as mean ± SD, and categorical variables are articulated as percentages. For continuous variables, statistical comparisons were conducted utilizing the *t* test, while categorical variables were evaluated using either Pearson's *χ* ^2^ test or Fisher's exact test, contingent upon appropriateness. Binary logistic regression analysis was deployed to probe the association between each determinant and CAD. The diagnostic accuracy of both HF‐QRS analysis and conventional ST‐segment analysis was evaluated by computing the sensitivity, specificity, and positive and negative likelihood ratio, using CAG results as the gold standard. The comparative analysis of the diagnostic methods was implemented using McNemar's test. Additionally, the correlation between HF‐QRS measurements and the severity of the disease was determined using Spearman's rank correlation coefficient. All statistical analyses were executed using SPSS 27.0 (IBM). Hypothesis testing was bidirectional, and a *p* value of less than .05 was considered statistically significant.

## 3 RESULTS

The study enrolled a total of 239 patients. Forty‐six patients were excluded from the analysis due to their inability to reach the target heart rate, corresponding to 80% of their MPHR. The assessment of HF‐QRS in 29 patients was hindered by inadequate signal quality. Moreover, two patients were excluded due to atrial fibrillation or atrial flutter, and an additional five patients were excluded due to a prolonged QRS duration of 120 ms or more. Consequently, the analysis included a total of 157 patients.

Table 1 delineates the clinical characteristics of the 157 patients included in the analysis, categorized based on their CAG results into two groups: those without CAD (stenosis less than 50%) and those with CAD (stenosis of 50% or more). Compared to patients without CAD, those diagnosed with CAD were predominantly male and exhibited a higher likelihood of being smokers. The CAD group also showed a higher prevalence of diabetes mellitus, hypertension, and a history of documented CAD.

**Table 1:** Clinical characteristics of study patients.

	No CAD (*n* = 67, 43%)	CAD (*n* = 90, 57%)	All (*N* = 157)	*p* Value
Age (years)	60 ± 11	63 ± 8	62 ± 9	0.077
Male	39 (58%)	71 (79%)	110 (70%)	**0.005**
BMI (kg/m^2^)	25.2 ± 3.6	24.5 ± 2.9	24.8 ± 3.2	0.233
Diabetes mellitus	11 (16%)	32 (36%)	43 (27%)	**0.008**
Hypertension	33 (49%)	62 (69%)	95 (61%)	**0.013**
Dyslipidemia	15 (22%)	22 (24%)	37 (24%)	0.764
Smokers	24 (36%)	48 (53%)	72 (46%)	**0.029**
History of CAD	16 (24%)	38 (42%)	54 (34%)	**0.017**
History of AMI	2 (3%)	10 (11%)	12 (8%)	0.058
Prior coronary revascularization	6 (9%)	15 (17%)	21 (13%)	0.160
Chest pain	34 (51%)	57 (63%)	91 (58%)	0.114
Abnormal repolarization in rest ECG	19 (28%)	30 (33%)	49 (31%)	0.506

*Note*: Bold values indicate statistically significant at p < 0.05.

Abbreviations: AMI, acute myocardial infarction; BMI, body mass index; CAD, coronary artery disease; ECG, electrocardiogram.

Table 2 summarizes the variables associated with the exercise test, ECG interpretation, and HF‐QRS analysis. Patients in the CAD group achieved a lower maximum heart rate, a lower percentile of their MPHR, and a slower postpeak recovery heart rate compared to the group without CAD. However, no significant differences were found in the resting or peak blood pressure. The positive rate of conventional st‐segment analysis in the CAD group was higher than that in the non‐CAD group, while only 39% of patients in the CAD group were positive. The number of leads with a positive HF‐QRS response, as well as the number of positive HF‐QRS tests (with three or more positive leads), was higher in the CAD group than in the no‐CAD group.

**Table 2:** Exercise, electrocardiographic, HF‐QRS, and angiography variables.

	No CAD (*n* = 67, 43%)	CAD (*n* = 90, 57%)	All (*N* = 157)	*p* Value
Exercise duration (s)	363 ± 26	368 ± 30	366 ± 28	.272
Resting heart rate (beats/min)	83 ± 18	82 ± 52	83 ± 41	.869
Max heart rate (beats/min)	150 ± 31	134 ± 34	141 ± 34	.004
Max heart rate (% MPHR)	86 ± 8	78 ± 10	81 ± 10	<.001
Postpeak recovery heart rate (beats/min)	136 ±17	117 ± 20	125 ± 21	<.001
Resting systolic blood pressure (mmHg)	134 ± 18	133 ± 22	134 ± 20	.772
Resting diastolic blood pressure (mmHg)	78 ± 13	78 ± 12	78 ± 12	.874
Peak systolic blood pressure (mmHg)	181 ± 23	173 ± 30	176 ± 28	.051
Peak diastolic blood pressure (mmHg)	89 ± 18	86 ± 13	87 ± 15	.308
Positive ST‐segment analysis	12 (18%)	35 (39%)	47 (30%)	.005
*HF‐QRS interpretation*				
Max power (uV)	7.9 ± 3.5	8.4 ± 2.8	8.2 ± 3.1	.368
QRS duration (ms)	95 ± 9	95 ± 8	95 ± 8	.705
Number of leads with positive HF‐QRS response	2.0 ± 2.7	4.2 ± 3.8	3.3 ± 3.6	<.001
Positive HF‐QRS tests	23 (34%)	57 (63%)	80 (51%)	.001

Abbreviations: CAD, coronary artery disease; HF‐QRS, high‐frequency QRS; MPHR, maximum age‐predicted heart rate.

In a multivariable binary logistic regression analysis, which adjusted for sex, hypertension, diabetes mellitus, smoking habits, history of CAD, and ST‐deviations, it was observed that there existed a positive correlation between HF‐QRS analysis and CAD. Importantly, HF‐QRS analysis was established as an independent predictor for the identification of CAD, with an odds ratio of 3.3 (95% confidence interval: 1.6–6.8, *p* = .001) (Table 3).

**Table 3:** Multivariable binary logistic regression analysis for the detection of CAD.

	OR	95% CI	*p* Value
Male	1.779	0.707–4.480	.221
Hypertension	2.325	1.094–4.940	.028
Diabetes mellitus	2.157	0.914–5.088	.079
Smokers	1.307	0.555–3.080	.540
History of CAD	1.857	0.847–4.069	.122
ST‐segment analysis	2.250	0.968–5.227	.059
HF‐QRS analysis	3.287	1.578–6.846	**.001**

*Note*: Bold value indicates that HF‐QRS analysis was established as an independent predictor for the identification of CAD.

Abbreviations: CAD, coronary artery disease; CI, confidence interval; HF‐QRS, high‐frequency QRS; OR, odds ratio.

Table 4 delineates the sensitivity, specificity, and positive and negative likelihood ratio of HF‐QRS analysis for CAD detection as 63%, 66%, 1.85, and 0.56, respectively. The HF‐QRS analysis demonstrated superior sensitivity (63% vs. 37%, *p* = .002), higher positive likelihood ratio (1.85 vs. 1.76) and lower negative likelihood ratio (0.56 vs. 0.80) in comparison to the conventional ST‐segment analysis for the detection of CAD. Nevertheless, conventional ST‐segment analysis exhibited higher specificity in detecting CAD (79% vs. 66%, *p* = .071) as shown in Table 4.

**Table 4:** Comparison of sensitivities and specificities of HF‐QRS analysis and ST‐segment analysis for the detection of CAD.

Detection of CAD	Sensitivity (*n* = 90)	Specificity (*n* = 67)	PLR	NLR
HF‐QRS analysis	63%	66%	1.85	0.56
ST‐segment analysis	37%	79%	1.76	0.80
*p* value for comparison HF‐QRS versus ST‐segment analysis	.002	.071		

Abbreviations: CAD, coronary artery disease; HF‐QRS, high‐frequency QRS; NLR, negative likelihood ratio; PLR, positive likelihood ratio.

The study incorporated both HF‐QRS analysis and ST‐segment analysis for CAD detection, employing serial test and parallel test methodologies. The diagnostic efficacy of the combined application of HF‐QRS analysis and ST‐segment analysis is summarized in Table 5. The sensitivity, specificity, and positive and negative likelihood ratios of the serial test were 26%, 97%, 8.67, and 0.76, respectively. Compared to ST‐deviation analysis alone, the serial test significantly increased specificity from 79% to 97% (*p* = .002). However, it showed a markedly low sensitivity of 26%. Correspondingly, the sensitivity, specificity, and positive and negative likelihood ratios of the parallel test were 77%, 51%, 1.57, and 0.45, respectively. Compared to ST‐deviation analysis alone, the parallel test significantly increased sensitivity from 37% to 77% (*p* < .001), while retaining a moderate level of specificity of 51%.

**Table 5:** Sensitivities and specificities of serial test and parallel test for the detection of CAD.

Detection of CAD	Sensitivity (*n* = 90), %	Specificity (*n* = 67), %	PLR	NLR
Serial test	26	97*	8.67	0.76
Parallel test	77**	51	1.57	0.45

Abbreviations: CAD, coronary artery disease; NLR, negative likelihood ratio; PLR, positive likelihood ratio

* *p* = .002 versus ST‐segment analysis.

** *p* < .001 versus ST‐segment analysis.

According to the results derived from CAG, the severity of CAD was represented by the extent of coronary artery stenosis (<50%, 50%–70%, and ≥70%) and the quantity of vascular lesions (with statistics for stenosis ≥50% or ≥70%). Supporting Information S1: Table 1 indicates that the number of leads exhibiting a positive HF‐QRS response is correlated with the severity of the disease.

## 4 DISCUSSION

This prospective investigation was designed to appraise the utility of HF‐QRS analysis in ascertaining the presence of CAD. This appraisal was conducted in juxtaposition with the conventional ST‐segment analysis, with CAG employed as the definitive standard for CAD confirmation. The main discoveries of the study are encapsulated as follows:

1. HF‐QRS analysis was identified as a statistically significant independent determinant for the detection of CAD (*p* = .001).
2. In comparison to conventional ST‐segment analysis, HF‐QRS analysis demonstrated superior sensitivity (63% vs. 37%, *p* = .002), higher positive likelihood ratio (1.85 vs. 1.76), and lower negative likelihood ratio (0.56 vs. 0.80) for the detection of CAD.
3. In relation to the detection of CAD, the serial test significantly increased specificity from 79% to 97% (*p* = .002) compared to ST‐deviation analysis alone. However, it showed a markedly low sensitivity of 26%. In contrast, the parallel test significantly increased sensitivity from 37% to 77% (*p* < .001) compared to ST‐deviation analysis alone, while retaining a moderate level of specificity of 51%.
4. A correlation was observed between the severity of CAD and the number of ECG leads presenting a positive HF‐QRS response.

The implications of our findings are substantial for clinical practice. Currently, the preliminary diagnosis and evaluation of patients with CAD predominantly rely on conventional resting or exercise ECG tests, with ST‐depression being the primary indicator of altered repolarization.^2^ However, the diagnostic accuracy of conventional ST‐segment analysis, particularly its sensitivity, is widely recognized as limited. Despite its cost‐effectiveness and noninvasive nature, its clinical utility in this context is progressively declining. Our study reiterates the previously reported inadequate sensitivity and specificity of conventional ST‐segment analysis.^3^, ^4^, ^5^, ^6^ In contrast, HF‐QRS analysis, which primarily focuses on depolarization abnormalities, manifested superior sensitivity, higher positive likelihood ratio, and lower negative likelihood ratio for CAD detection compared to conventional ST‐segment analysis in our study. This aligns with previous reports on the performance of HF‐QRS, which used MPI as the gold standard for CAD.^18^, ^19^, ^20^, ^21^ By employing the anatomical gold standards provided by CAG, our study corroborates and expands upon previous studies, affirming that HF‐QRS analysis is superior to conventional ST‐segment analysis in detecting CAD.

This study demonstrates the potential to enhance the relatively modest diagnostic accuracy of EST for CAD detection by combining HF‐QRS and conventional ST‐segment analysis through serial or parallel tests. In our study, compared to ST‐deviation analysis alone, the serial test enhanced specificity from 79% to 97% (*p* = .002), while the parallel test amplified sensitivity from 37% to 77% (*p* < .001). However, the serial test showed a markedly low sensitivity of 26%, indicating a high rate of missed diagnoses and limiting its clinical utility. The parallel test demonstrated both high sensitivity (77%) and maintained a moderate level of specificity (51%) in comparison. Therefore, compared with the serial test, the parallel test may have greater clinical value. This finding aligns with results from a large‐scale study led by Professor Schaerli et al.,^15^ where they employed a combined algorithm. This algorithm categorized patients as “rule‐out” (both tests negative), “rule‐in” (both tests positive), and an intermediate zone (only one test positive). Their study demonstrated improved sensitivity for “rule‐out” and specificity for “rule‐in” at 63% and 97%, respectively, compared to ST‐deviation analysis alone. The algorithm could assist patients in the “rule‐in” and “rule‐out” groups, but those in the intermediate zone do not derive direct benefits from it. Our study utilized a distinct approach to combine HF‐QRS and conventional ST‐segment analysis, employing different gold standards for CAD detection, yet achieved similar results. Importantly, our approach has the potential to benefit all patients, thus corroborating and expanding upon the previous study.

Indices derived from HF‐QRS provided information about the severity of CAD, as the number of leads with positive HF‐QRS response was correlated to the extent of coronary artery stenosis (<50%, 50%–70%, and ≥70%) and the quantity of vascular lesions (with statistics for stenosis ≥50% or ≥70%). This observation is consistent with earlier studies that have established a correlation between the quantity of positive HF‐QRS leads and the extent of MPI ischemia or the number of occluded coronary vessels.^14^, ^22^

Conventional ST‐segment analysis necessitates the expertise of professional electrocardiologists for result interpretation, a process susceptible to individual variations in professional proficiency. In contrast, outcomes from HF‐QRS analysis are exclusively generated via automated computer algorithms, thereby rendering them inherently more objective, rapid, and precise. As a result, HF‐QRS analysis may hold greater potential value in the diagnosis of CAD.

## 5 LIMITATIONS

Several constraints are inherent in this investigation and merit due consideration. Initially, the study was limited by the relatively diminutive size of the patient cohort analyzed. This is primarily attributed to the prevalent elderly demographic typically subjected to stress testing, wherein the attainment of 80% of the MPHR can present considerable challenges, thus leading to their exclusion from the analysis. In our study, the proportion of patients who achieved this threshold was merely 19%. Subsequently, HF‐QRS analysis encounters a constraint in the form of a high incidence of non‐diagnostic test outcomes. This is partially attributable to the technique's dependency on a high‐quality acquired ECG signal, which is indispensable for the detection of subtle microvolt alterations.^23^ Enhanced adherence to patient preparation protocols and conventions for electrode attachment could substantially alleviate this issue, a consideration worthy of incorporation in future research. Thirdly, a significant fraction, amounting to one‐third of the study population, had a known history of CAD, and some had even undergone coronary revascularization. Notwithstanding this, the diagnostic efficacy of HF‐QRS in detecting CAD remained consistent within these patients. Lastly, in certain instances, β‐blockers or vasodilator medications were not discontinued before the test, potentially introducing a confounding variable that might influence the study outcomes. Despite this potential influence, the study yielded significant insights. For the optimal diagnostic performance of HF‐QRS analysis for CAD, strict discontinuation of medications before testing is advocated.

## 6 CONCLUSION

In summation, the incorporation of HF‐QRS analysis during exercise tests manifested a superior sensitivity in detecting angiographically confirmed CAD in comparison to conventional ST‐segment analysis. Moreover, the combination of HF‐QRS and ST changes during exercise tests exhibited an enhanced diagnostic performance for CAD.

## CONFLICT OF INTEREST STATEMENT

The authors declare no conflict of interest.

## Supporting information

Supporting information.

## Data Availability

The data that support the findings of this study are available on request from the corresponding author. The data are not publicly available due to privacy or ethical restrictions.
