# Supplementary material for: The combination of high‐frequency QRS and ST‐segment alterations during exercise stress tests enhanced the diagnostic efficacy for coronary artery disease
Source: Clin Cardiol. 2024 Mar 13;47(3):e24254. doi: 10.1002/clc.24254 (PMC10933670; doi:10.1002/clc.24254)
Supplement: Supplementary file 1 — Supporting information. [file CLC-47-e24254-s001.docx]

**Supporting information**

**Supplementary Figure 1.** Principles of high-frequency QRS (HF-QRS) analysis.


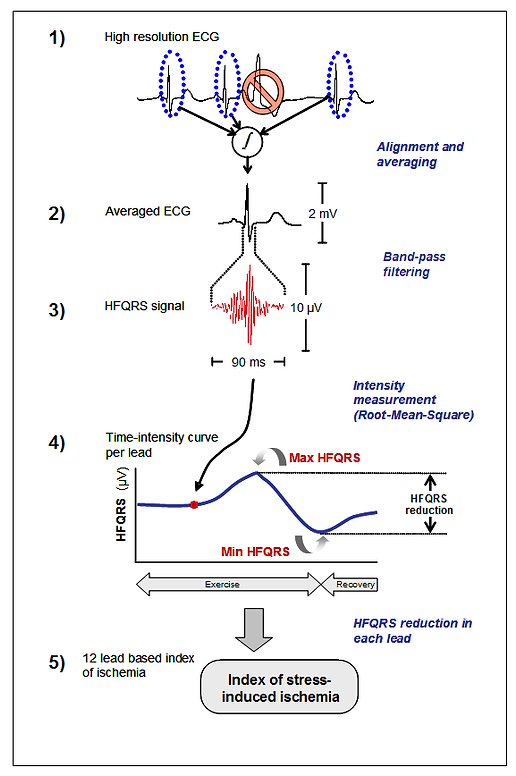


**1)** Employing cross-correlation techniques, QRS complexes are identified, with the exclusion of arrhythmias and noisy complexes. **2)** The identified QRS complexes are aligned and averaged, minimizing noise within a 150-second sliding window incremented at 10-second intervals. **3)** Filtering within the 150 to 250 Hz frequency band generates the HF-QRS signal at a specific time point. **4)** The HF-QRS time-intensity curve is computed for each lead throughout the entire exercise test. **5)** Ischemia index is determined by assessing the reduction in HF-QRS intensity across all 12 leads.

**Supplementary Table 1**. Correlation between HF-QRS response and severity of CAD at angiography.

|  | ρ | p-value |
| --- | --- | --- |
| The extent of coronary artery stenosis | 0.38 | **<0.001** |
| The number of vascular lesions (stenosis ≥50%) | 0.38 | **<0.001** |
| The number of vascular lesions (stenosis ≥70%) | **0.41** | **<0.001** |

The number of leads with significant HFQRS intensity reduction vs. the extent of coronary occlusion and the number of vessels with obstructive lesions.

CAD: coronary artery disease; HF-QRS: high frequency QRS
